# Supplementary material for: Gas Chromatography-Mass Spectrometry and Single Nucleotide Polymorphism-Genotype-By-Sequencing Analyses Reveal the Bean Chemical Profiles and Relatedness of Coffea canephora Genotypes in Nigeria
Source: Plants (Basel). 2019 Oct 18;8(10):425. doi: 10.3390/plants8100425 (PMC6843358; doi:10.3390/plants8100425)
Supplement: Supplementary file 1 [file plants-08-00425-s001.pdf]

plants

MDPI

Supplemental Information

Gas Chromatography-Mass Spectrometry and Single Nucleotide Polymorphism-Genotype-By-Sequencing reveal the chemotypes of *C. canephora* genotypes Nigeria

Supplementary Table 1: The eight classes of metabolites identified in the Nigerian *C. canephora* coffee genotypes. Metabolite classes include amines, amino acids, fatty acid, organic/inorganic compounds, polyphenol, sugar derivatives, sugar and vitamins. Metabolites in bold have the highest concentration within each of the classes. Metabolites in the top rows within a class are most abundant, and those in the bottom row are least abundant.

**Amines**			
**Putrescine**	Tyramine	Urea	Uric acid
Uridine	Guanosine	Pseudo uridine	Maleimide
Hydroxylamine			
**Amino acids**			
**Aspartic acid**	Glutamic acid	Proline	Tryptophan
Phenylalanine	Homoserine	Cysteine	Trans-4-hydroxyproline
Citrulline	Tyrosine	Isoleucine	Beta-alanine
Alanine	Glycine	Methionine	Valine
Leucine	Beta-glutamic acid	N-acetyl-D-galactosamine	Serine
Histidine	Threonine	Cyanoalanine	Asparagine
Lysine	Glutamine	Oxoproline	
**Fatty acid**			
**Stearic acid**	Glycerol	Palmitic acid	Linoleic acid
Pelargonic acid	Lactic acid	Oleic acid	Cis-gondoic acid
Arachidic acid	Lauric acid	Isohexonic acid	Stigmasterol
Capric acid	Hexadecylglycerol	Nonadecanoic acid	Lignoceric acid
1-monoolein	1-monopalmitin		
**Organic/inorganic compounds**			
**Citric acid**	Malic acid	Fumaric acid	Maleic acid
2-hydroxyglutaric	Alpha-ketoglutarate	Lithocholic acid	Allantoic acid
5-hydroxy-3-indole	Isocitric acid	Aconitic acid	2-deoxytetronic acid

D-erthro-sphingos	Adipic acid	Succinic acid	Shikimic acid
Phosphate	Pipecolinic acid	Alpha-aminoadipic	4-aminobutyric acid
Benzoic acid	3,4-dihydroxyhydrocinnamic acid NIST	3-hydroxybenzoic acid	Digalacturonic acid
Itaconic acid	Propane-1,3-diol	Vanillic acid	Glucosaminic acid
Citramalic acid	3,4-dihydroxybenzoate	Tartaric acid	4-hydroxybenzoate
Oxalic acid			
**Phenolic acids/Alkaloids**			
**Caffeine**	Chlorogenic acid	Quinic acid	3,4-dihydroxy-cinnamic acid
Gluconic acid	Ferulic acid	Gluconic acid lactone	Beta-sitosterol
Tocopherol beta NIST	Isochlorogenic acid	Tyrosol	Nornicotine
**Sugar derivatives**			
**Galactinol**	5-methoxytryptamine	Saccharic acid	Glycerol-3-galactoside
6-deoxyglucitol	Mannitol	1-methylgalactose	Butane-2,3-diol NIST
Lactobionic acid	3,6-andro-D-galactose	Glucose-1-phosphate	1,2-andro-myo-inositol
Ribonic acid	Catechinflavan-3-ol	Methanolphosphate	5-hydroxynorvaline NIST
Conduritol-beta-epoxide	2-monoolein	1-monostearin	Galactitol
Galactonic acid	Maltitol	Hexitol	Hydroquinoaromatic
Arbutin	Lactitol	4′,5-dihydroxy-7-glucosyloxyflavanone	Threonic acid
Glycolic acid	6-deoxyglucose	2-monostearin NIST	Butyrolactam NIST
Glycerol-alpha-phosphate	Lyxitol	Arabitol	UDP-glucuronic acid
Isothreonic acid	Glyceric acid	Erythritol	Mucic acid
**Sugars**			
**Sucrose**	Fructose	Glucose	Galactose
Sophorose	Threitol	Palatinitol	Sorbitol
Pentitol	Inulotriose	Melezitose	Tagatose
Raffinose	N-acetyl-D-mannosamine	Beta-gentiobiose	Fucose
Xylose	Trisaccharide	Mannose	

**Vitamins**		
**Myo-inositol**	Nicotinic acid (Vit B3) or Niacin	Inositol-4-monophosphate

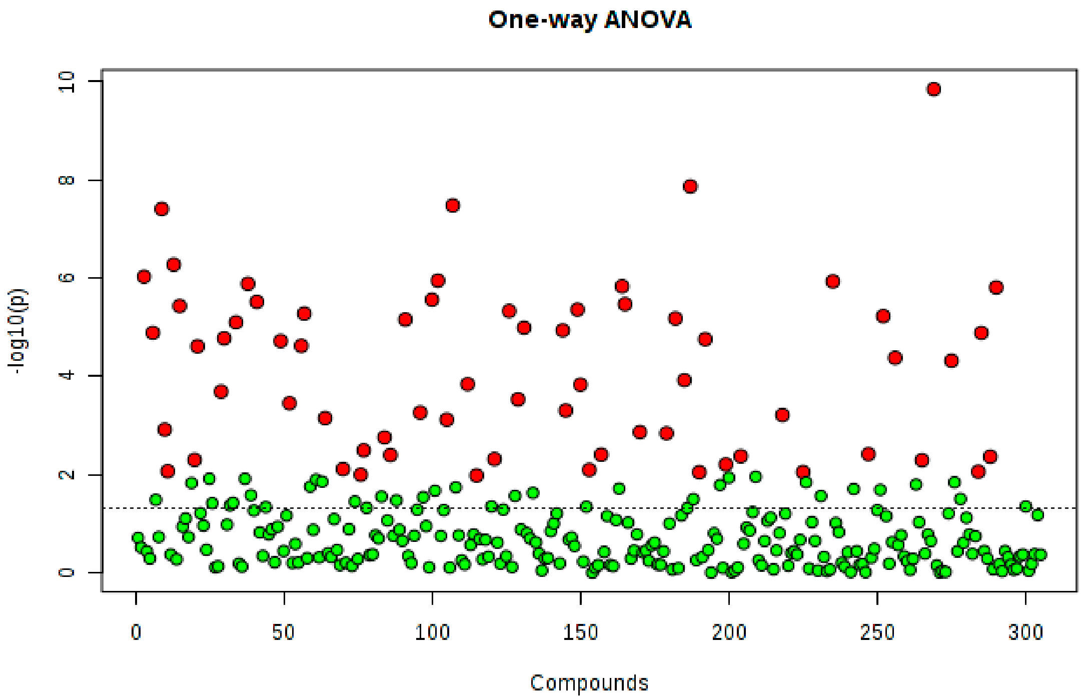

Figure S1. **Metabolites detected by One-Way Analysis of variation that significantly varied across genotypes.** With the statistical significant level cut-off at 2 (p=0.01), there were 66 metabolites (red circles) that met this criteria.

Supplementary Table 2: Fatty acids with high Pearson's Coefficient correlative scores (r^2>0.80; p<0.05)

	Glycerol	Linoleic acid	Arachidic acid	Stearic acid	Palmitic acid
Glycerol	1	0.89409	0.83245	0.80049	0.88363
Linoleic acid	0.89409	1	0.86853	0.88823	0.91323
Arachidic acid	0.83245	0.86853	1	0.82577	0.85591
Stearic acid	0.80049	0.88823	0.82577	1	0.89874
Palmitic acid	0.88363	0.91323	0.85591	0.89874	1
